# The intrinsic plasticity of medial vestibular nucleus neurons during vestibular compensation—a systematic review and meta-analysis

**DOI:** 10.1186/s13643-020-01399-2

**Published:** 2020-06-17

**Authors:** Rajiv Wijesinghe, Aaron Camp

**Affiliations:** Sensory systems and integration laboratory, Sydney Medical School, University of Sydney, Sydney, NSW 2006 Australia

**Keywords:** Vestibular compensation, UVD, Intrinsic plasticity, Medial vestibular nucleus

## Abstract

**Background:**

Vestibular compensation is a homeostatic process that occurs in the central nervous system in response to peripheral vestibular dysfunction. Experimental studies in rodent models have suggested that unilateral peripheral vestibular lesions are correlated with an increase in the intrinsic excitability of central vestibular neurons. This process may be dependent on the intrinsic properties of the neurons themselves. We aimed to conduct a systematic review of the literature to survey the evidence for changes in intrinsic plasticity observed during the acute phase of vestibular compensation.

**Methods:**

We systematically reviewed the literature regarding the electrophysiological effect of experimentally induced unilateral vestibular deafferentation (UVD) on the intrinsic membrane properties of medial vestibular nucleus neurons in animal models. We developed tools to assess the methodological quality (precision, validity and bias) of studies that met pre-determined inclusion and exclusion criteria. We extracted numerical data and performed a meta-analysis of specific quantitative data pooled from these studies.

**Results:**

We identified 17 studies that satisfied the inclusion criteria. There is moderate quality evidence to suggest a statistically significant increase in the intrinsic excitability of medial vestibular nucleus neurons following unilateral vestibular deafferentation. Specifically, the spontaneous discharge rate increases by 4 spikes/s on average and the sensitivity to current stimuli increases.

**Conclusion:**

Using this novel approach, we demonstrate that the methodology of systematic review and meta-analysis is a useful tool in the summation of data across experimental animal studies with similar aims.

## Background

The vestibular system acts to detect changes in head position and maintain our sense of equilibrium unconsciously. The sensory information used by this system is derived from the paired vestibular organs, visual inputs and sensory and proprioceptive feedback from the limbs. These signals are received by a complex of vestibular nuclei within the brainstem and distributed to brainstem oculomotor and spinal locomotor effector circuits. This integrated neural network mediates postural control when stationary, maintains gaze stability to create a stable visual world during movement and generates our perception of orientation and motion in space.

Peripheral vestibular dysfunction can be caused by a number of disease processes, including viral illnesses (vestibular neuritis), antibiotic toxicity (aminoglycosides) or may be iatrogenic (vestibular neurectomy for refractory vertigo). Vestibular dysfunction is characterised by distinct oculomotor and postural deficits that are observed in human subjects [1] and can be induced experimentally in animal models [2]. These deficits are static (present when there is no applied stimulus apart from gravity) or dynamic (revealed by movement). Static deficits observed in humans include the symptom of vertigo, a postural bias towards the lesioned side, spontaneous nystagmus (SN) with a slow phase to the affected side and the ocular tilt reaction [3]. When unilateral vestibular deafferentation (UVD) is induced experimentally in animal models, the intensity of these deficits abates over days, such that they may only be revealed in certain circumstances [3]. For example, SN typically disappears after by one week in guinea pigs [4], rodents [5], cats [6], monkeys [7] and humans [8]. Static deficits have been shown to recover in the absence of visual and cerebellar [9] inputs, consistent with the idea that this is a purely vestibular phenomenon.

The recovery of these behaviours could be due to either a restoration of vestibular function or a substitution of analogous non-vestibular sensory information. Early physiological changes appear to be insufficient to reconstitute dynamic vestibular function, which remains asymmetrical and ineffective [10] without extra-vestibular substitutions [11]. However, the basic function of the vestibuloocular reflex (VOR) does seem to recover and is therefore a useful tool to understand the physiological changes that restore static deficits [12]. The VOR is dependent on a balance of activity between the paired vestibular nuclei, as head movement is encoded by changes in tonic afferent discharge depending on the direction of movement. Therefore, one would expect that for vestibular function to be restored to normal, discharge in the deafferented nucleus would remain tonically active, even if not at baseline levels.

Immediately following experimental UVD in animals, in vivo recordings demonstrate a decrease in the proportion of spontaneously active medial vestibular nucleus (MVN) neurons, as well as a reduction in their spontaneous discharge rate. However, within hours to days for most studied models, the number of recordable neurons increases and their discharge rate increases. This occurs despite being deprived of a large amount of sensory afferent input and the presence of inhibitory projections from the intact side [13]. This observation suggests that the return of vestibular neuron activity is intrinsic to the neuron itself and external influences may be insufficient to explain this recovery. This intrinsic mechanism hypothesis [12] posits that at least part of the process of vestibular compensation is a manifestation of experience-dependent intrinsic plasticity. A number of in vitro studies to date have demonstrated an increase in the spontaneous discharge rate of vestibular neurons during this process [14–17]; however, the magnitude of this change remains unclear [18, 19]. We performed a systematic search of the published literature to identify primary sources of information examining the effects of unilateral vestibular deafferentation on the intrinsic membrane properties of medial vestibular nucleus neurons during the acute period of vestibular compensation in animal models. Expressed in the PICOS format:

- Participants—medial vestibular nucleus neurons following unilateral vestibular deafferentation
- Interventions—unilateral vestibular deafferentation
- Controls—medial vestibular nucleus neurons in animals with an intact vestibular system
- Outcomes—changes in characteristics of intrinsic membrane properties
- Study designs—animal research electrophysiological studies

We extracted quantitative data from these sources and performed a meta-analysis to estimate the magnitude of the effect of UVD.

## Methods

To reduce the risk of bias, the Cochrane Collaboration guidelines were used to perform literature searches for the systematic review [20]. The results of the systematic review are reported using criteria adapted from the Preferred Reporting Items for Systematic Reviews and Meta-Analyses (PRISMA) statement [21]. While not pre-registered, the review was performed within the guidelines of a pre-determined study protocol and has been made available at Open Science Framework (https://osf.io/gbd6m).

### Search strategy

The following electronic databases were searched for relevant studies relating to the study question: MEDLINE (OvidSP), 1 January 1946 to 1 October 2017; Pubmed (Internet), up to 1 October 2017; and Embase (Internet), up to 1 October 2017. Searches were not limited by publication date, language or publication status at the time of search. The final search was performed on 2 October 2017. The search was repeated on 6 January 2020 and no new relevant articles were identified. The references within all included studies or narrative reviews were hand searched to identify any further studies that may have satisfied the inclusion criteria. Titles and abstracts from the final search were pooled into an Endnote® database and duplicates were removed. Each reference was subjected to the above inclusion criteria by two assessors independently and any conflicts were mediated through discussion. Following this, the full texts of articles were obtained and assessed rigorously to ensure they satisfied the inclusion criteria. At this stage, articles not in English or containing previously published data were removed. Studies were identified by the surname of the first author and the year of publication. The following is an example of the search strategy used to search the MEDLINE database: 1 AND (2 or 3 or 4 or 5 or 6) AND (7 or 8 or 9 or 10 or 11)

1. medial vestibular.mp or MVN.mp
2. labyrinthectomy.mp or labyrinthectom*
3. deafferentation.mp or deafferent*
4. neurectomy.mp
5. lesion*
6. compensation.mp
7. intrinsic.mp
8. ion channels/ or ion channel*.mp
9. Action Potentials/ or action potential*.mp
10. Membrane Potentials/ or membrane potential*.mp
11. excitability.mp

### Definitions

We defined intrinsic excitability as the neuronal activity determined by structural features of the cell membrane, ion channel expression and intracellular buffering proteins that control concentrations of ions. We restricted analysis to active membrane properties that shape incoming inputs and maintain activity in the absence of synaptic input. These properties are reflected by the rate of spontaneous firing, or by the response to graded current stimuli (also known as the gain). Vestibular compensation is the gradual restoration of vestibular function following damage to the vestibular system [22]. In animal models, the behaviours observed are changes in posturing, spontaneous nystagmus and abnormal turning and rolling behaviours. This process may be acute (less than 2 weeks) or chronic (greater than 2 weeks). The animal model widely used to study vestibular compensation is experimentally induced UVD [2, 23].

### Inclusion criteria

Studies were included if they presented original research examining intrinsic neuronal properties during the process of vestibular compensation. Reviews, abstracts, conference proceedings, commentaries and non-English articles were excluded. Analysis was restricted to adult (i.e. mature) animal models. Vestibular lesions could be performed by any method, including physical or chemical disruption of the vestibular labyrinth, vestibular nerve transection or focal ototoxic drug administration. From herein UVD will refer to these lesions collectively. Ideally, the success of the deafferentation procedure was confirmed with behavioural or anatomical observations; however, this was not a requisite for inclusion. Intrinsic properties could be studied using direct intracellular patch clamp or extracellular whole cell recordings of visually identified MVN neurons in vitro. Studies that inferred intrinsic properties through protein expression changes of ion channels or intracellular electrolyte buffers were also included.

For the meta-analysis, studies were restricted to intracellular patch clamp recordings taken from MVN neurons. Extracellular whole cell recordings were also included if, within the study itself, differences between intracellular recordings were justified as similar. Studies presenting gain measurements were included if, within the presented data, information regarding the raw spontaneous firing rates was explicit or able to be derived. Studies that performed electrophysiological recordings but did not account for blockade of synaptic currents were excluded. Studies included in the systematic review and meta-analysis are listed in Table 1.

**Table 1:** Studies included in systematic review

Study	Animal model	Duration of compensation	Recording type	Sample size	Outcome measure	Notable features
de Waele 1994 [24]	Rat, UL via ischaemia (photocoagulation of the pterygopalatine artery) and mechanical disruption, compared to unoperated control	5 h 3 days 3 weeks	Visual identification of cells labelled with antisense oligonucleotides against NMDA or metabotropic glutamate receptor	Control—6 animals 5 h—6 animals 3 days—unclear, possibly 3 animals 3 weeks—6 animals	Number of silver grains per cell, silver grain density in cells	Success of UL was assessed behaviourally; however, the number of excluded animals was not reported. There was inconsistent reporting animal numbers per experimental group in the methods and results.
Cameron 1997 [15](*)	Rat, UL via 100% ethanol injection, compared to sham-operated control	2 h 4 h 6 h 1 day 2 days	(Presumed) Extracellular recordings, ipsilesional	Control—5 animals, 2 sham-operated, number undergoing UL unclear 4 h—32 cells 6 h—43 cells 24 h—36 cells 2 h—unclear 48 h—unclear	Mean spontaneous discharge rate (spikes/s)	Sprague-Dawley rats used for experimentation were at the lower end of maturity (60–120 g) raising concerns about developmental stage. There was selective reporting of outcomes, with 2 and 48 h rates reported graphically rather than numerically.
Cameron 1999 [16](*)	Rat, UL via 100% ethanol injection, compared to sham-operated control	4 h 6 h	Single unit extracellular recordings, ipsilesional	Control—9 sham-operated, 41 neurons UL—9 animals, 61 neurons	Mean spontaneous discharge rate (spikes/s)	The type of animal and their baseline characteristics such as sex and age were not stated in the report. Additional interventions were studied—no withdrawal of anaesthesia following labyrinthectomy, and administration of dexamethasone or dexamethasone antagonist.
Vibert 1999 [25]	Guinea pig, UL via mechanical disruption, compared to unoperated control	1 day 3-7 days (pooled following analysis)	Single unit extracellular recordings and field potentials, ipsilesional and contralesional	Total of 65 animals 1 day—14 animals 3 days—26 animals 5 days—10 animals 7 days—15 animals	Mean spontaneous discharge rate (spikes/s), evoked field potentials following stimulation of the vestibular and abducens nerves	An unclear number of animals were excluded the stated reason being the difficulty of the surgical procedure yielding ‘unusable data’.
Yamanaka 2000 [14] (*)	Rat, UL via ethanol injection, compared to unoperated control	4 h	Extracellular whole cell, ipsilesional	Control—28 animals, 77 cells 4 h—44 animals, 126 cells	Mean spontaneous discharge rate (spikes/s), dose of GABA agonist required to inhibit tonic resting activity	Rostral and caudal MVN neurons were analysed separately in the study and therefore analysed separately in the meta-analysis.
Him 2001 [18] (*)	Rat, UL via 100% ethanol injection, compared to normal	1-3 days 7-10 days	Intracellular patch clamp, ipsilesional	Control—44 animals 1-3 day—34 animals, 90 cells 7-10 day—25 animals, 84 cells	Mean spontaneous discharge rate (spikes/s), action potential morphology	Inclusion criteria are mentioned however never explicitly stated. Some data is lost without being accounted for.
Johnston 2001 [26] (*)	Rat, UL via 100% ethanol injection, compared to normal	4 h 1 day 2 days 7-10 days	Single unit extracellular recordings, ipsilesional	Control—25 cells 2 days—42 cells 7-10 days—40 cells Unclear how many animals in control group or underwent UL	Mean spontaneous discharge rate (spikes/s)	Control, 4 and 24 h data was obtained from a previous study and therefore not included in the current set of data.
Ris 2001 (*)	Guinea pig, UL via mechanical disruption, compared to normal	2 days 7 days	Single unit extracellular recordings, ipsilesional	Control—10 animals, 57 cells 2 days—6 animals, 118 cells 7-10 days—6 animals, 159 cells	Number of spontaneously active neurons, mean spontaneous discharge rate (spikes/s)	A potentially ototoxic antibiotic was given during the surgical procedure (aminoglycoside class) however such a short duration of exposure is unlikely to have any significant effect.
Johnston 2002 [27]	Rat, UL via 100% ethanol injection, compared to normal and flocculectomised animals	4 h 2 days	Single unit extracellular recordings, ipsilesional	Control—5 animals, 189 cells 4 h—5 animals, 187 cells 2 days—5 animals, 126 cells	Mean spontaneous discharge rate (spikes/s)	Numerical data could not be extracted in sufficient detail as it was presented graphically. Therefore, it was excluded from the meta-analysis.
Ris 2002 [28]	Guinea pig, UL via mechanical disruption and ototoxic antibiotic injection, compared to normal	7 days	Intracellular patch clamp recordings, ipsilesional	Control—38 cells 7 days—38 cells	Gain (spikes/s/nA), dynamic responsiveness (maximal firing rate compared to steady-state rate)	Unclear how many animals were used in the experiment. All recordings in the presence of synaptic antagonists, held from a hyperpolarised membrane potential to abolish spontaneous discharge.
Patko 2003 [29]	Rats, UL by mechanical disruption, compared to unoperated controls	1 day 3 days 8 days 30 days	In situ hybridisation using mRNA probes to sodium and potassium channels	Total—42 rats 3 rats for each channel and time point	Optical density of labelling	There was no evidence to suggest changes in the abundance of these channels to explain the observed changes in excitability.
Ris 2003 [30]	Guinea pig, UL via mechanical disruption and ototoxic antibiotic injection, compared to normal	7 days	Intracellular patch clamp recordings, ipsilesional; immunohistochemistry labelling calcium channel proteins	Control—28 animals, 41 cells 7 days—21 animals, 43 cells	Spike discharge characteristics; changes in immunohistochemical staining patterns	An increase in low threshold spiking type activity was observed in all neurons, without a detectable change in the immunolabelling of protein subunits of T-type calcium channels.
Eleore 2004	Rat, UL via mechanical disruption, compared to unoperated control	5 h 1 day 3 days 8 days 1 month 2 months	In situ hybridisation using mRNA probes to glycine receptor subunits and an anchor protein gephyrin, detected using autoradiography or immunofluorescence	Total—60 rats Control—12 animals, 6 used in final analysis Experimental—48 animals, 36 used in final analysis, with 3 animals per experimental group	Optical density of labelling; intensity of immunofluorescence	The discrepancy between the original number of animals included in each group and the number used in the final analysis is presumed to be due to exclusion because of failure to compensate post labyrinthectomy; however, this is not explicitly stated.
Guilding 2004	Rat, UL via 100% ethanol injection, compared to sham-operated control	4 h	Thin-layer chromatography quantified by optical densitometry	Control—16 animals 4 h—16 animals	Change in density on chromatography	No differences in the expression of an enzyme involved in the production of glucocorticoids following labyrinthectomy.
Guilding 2005 [31]	Rat, UL via ethanol injection, compared to unoperated control	4 h 2 days 7 days	Extracellular whole cell recordings, ipsilesional, before and after application of a cocktail of neurotransmitter antagonists (for blockade of GABA_A_, GABA_B_, glycine, AMPA and NMDA receptors)	Unclear how many animals were used in experiment control—85 cells, 75 cells synaptic blockade 4 h—76 cells, 70 cells synaptic blockade 48 h—82 cells, 80 cells synaptic blockade 7 days—90 cells, 90 cells synaptic blockade	Mean spontaneous discharge rate (spikes/s)	Rostral and caudal MVN neurons were analysed separately in the study. Numerical data could not be extracted in sufficient detail from the study for inclusion in the meta-analysis as it was presented graphically.
Nelson 2017 [19]	Mouse, UL via mechanical disruption, compared to sham-operated control	1 day 3 days 1 week 3 weeks	Intracellular patch clamp, ipsilesional; ion channel protein expression; ocular videography	At least 6 animals 8 h -140 control, 156 UL cells 1 day—142 control, 131 UL cells 3 days—91 control, 112 UL cells 7 days—96 control, 105 UL cells 21 days—63 control, 62 UL cells	Mean spontaneous discharge rate (spikes/s), gain (spikes/s/nA), firing rate potentiation	This was the only study to blind intervention allocation from investigators. It was unclear how many animals were used throughout the experiment and how many were in each experimental group.

Asterisked studies were included in the meta-analysis. *AMPA* α-amino-3-hydroxy-5-methyl-4-isoxazolepropionic acid, *C* control, *GABA* γ-aminobutyric acid, *NMDA N*-methyl-d-aspartate, *UL* unilateral labyrinthectomy

### Exclusion criteria

Studies of immature animals were excluded, as changes seen in younger animals may represent developmental modifications, rather than plastic changes that may occur at maturity. The effects of chronic lesions (>2 weeks) were also excluded, as changes after this time period may not reflect the early behavioural recovery of static deficits. Also, alternative complex network processes are believed to govern this chronic period of compensation [3]. Non-English articles were excluded as the team did not have the capacity to analyse data from papers published in any other languages.

### Assessment of methodological quality

Each study in this review was assessed for scientific precision, criterion validity (of the model) and risk of bias. The risk of bias for each study was assessed using the Systematic Review Centre for Laboratory Animal Experimentation (SYRCLE) tool which has been validated for use in animal studies [32]. For the assessment of validity and precision, published tools were not strictly applicable. Therefore, we developed criteria to assess these methodological domains. For each included study, questions were used by the reviewers to assign a rating based on how well the criterion was satisfied. If the answer was unequivocal, the relevant criterion was scored either as yes or no. If it was unclear, then the criterion was scored unclear. Often an unclear rating was assigned due to a lack of an explicit statement of necessary detail to answer the question, or ambiguous and vague descriptions within the published report.

#### Model validity

This tool assessed reporting of details of the animal model, its routine handling and the control used for experiments in each study. Model validity ratings are presented in Table 2. The following questions were asked of each study:

1. Was an ethical statement provided for animal handling and the use of biological tissue?
2. Were there clear descriptions of the model used to study vestibular compensation?
3. Was there a clear description of the routine maintenance of the model during experimentation?
4. Were details provided of how the model was prepared for the experimentation?
5. Did the authors prove the success of deafferentation?
6. Was there an appropriate and comparable control?

**Table 2:**
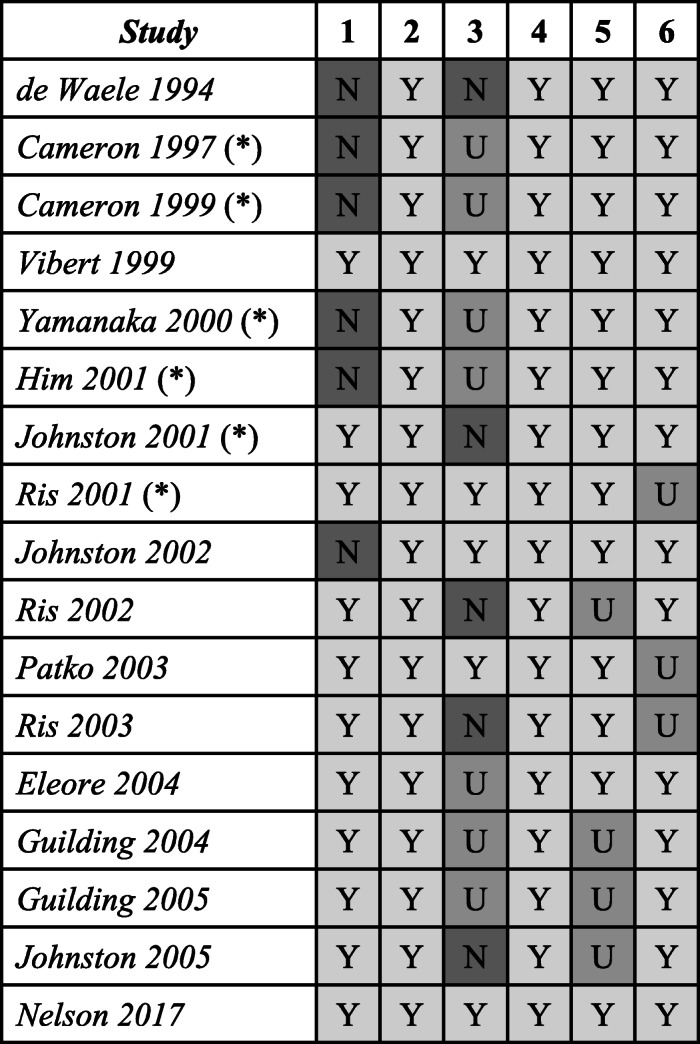
Ratings for domains assessing the validity of the model in studies included in the systematic review

Asterisked studies were included in the meta-analysis. *N* not satisfied, *U* unclear or insufficient evidence to make an assessment, *Y* criterion satisfied

#### Precision

This tool assessed the reporting of technical details of experimental structure, statistical methods used to analyse the significance of data and sample sizes. Some of these particular assessments (for example, calculation of sample sizes to achieve an appropriately powered study) are not routine in such non-clinical experimental work, however, do affect the precision of the conclusions made. Over and above the SYRCLE risk of bias tool, this specifically assessed for outlying data to make a valid assessment of the heterogeneity of data. Precision ratings are presented in Table 3. The following questions were asked of each study:

1. Were repeats of experiments performed per animal? This is referred to as technical variability
2. Did the experiment give the same result when it was repeated in a different animal? This is referred to as observer variability
3. Is it clear whether repeatability is a combination of technical and observer variability? I.e. where these two repeats reported individually or as a pooled result?
4. Did the result include an appropriate measure of variability?
5. Did the authors pool data from previous experiments? If so, did they assess for heterogeneity between experiments?
6. Were sample sizes required for significance calculated prior to experiments being conducted?
7. Were indeterminate, missing or outlying results handled appropriately?
8. Was the study appropriately powered to reach statistical significance?
9. Was there a clear statement or description of the statistical method?
10. Was the chosen statistical method appropriate?
11. Was there any evidence of data dredging?

**Table 3:**
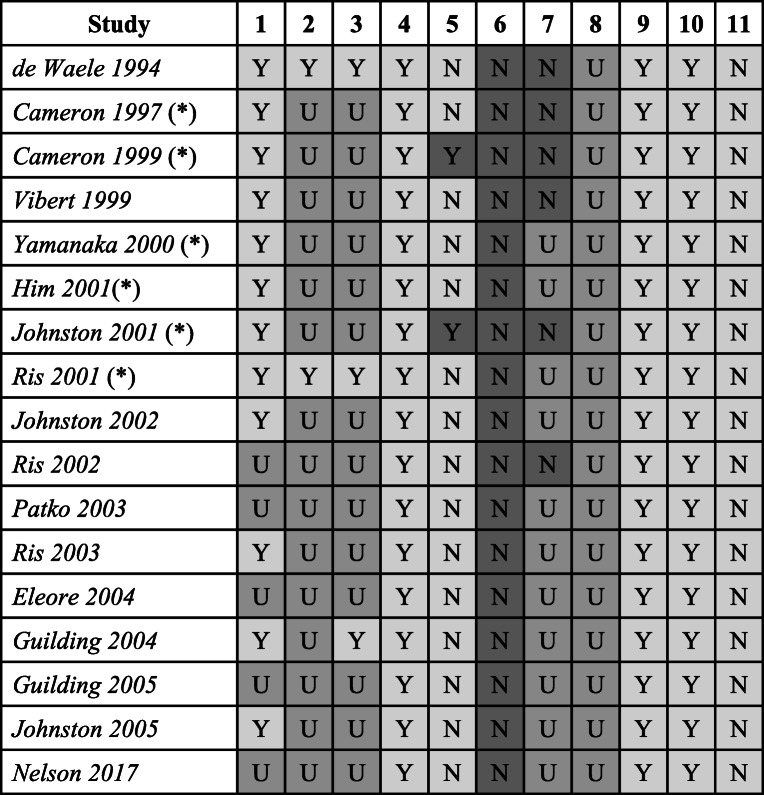
Ratings for domains assessing imprecision in included studies

*N* not satisfied, *U* unclear or insufficient evidence to make an assessment, *Y* criterion satisfied

#### Risk of bias

This tool is adapted from the SYRCLE risk of bias tool, which is modelled along the criteria used to assess human trial data [32]. It should be noted that many of the criteria listed below are still not routine for animal studies (for example, random allocation or sequence generation) and this was taken into account when assessing risk of bias. Risk of bias ratings are presented in Table 4. The following questions were asked of each study:

1. Were participants allocated randomly to experimental and control groups? If so, was this sequence adequately generated and applied?
2. Were the groups similar at baseline or were they adjusted for confounders in the analysis? The baseline characteristics considered to be important were the age of animal, sex of animal and housing arrangements.
3. Was the allocation adequately concealed?
4. Were the animals randomly housed during the experiment?
5. Were the caregivers and/or investigators blinded from knowledge of which intervention each animal received during the experiment? This is also known as allocation concealment.
6. Were animals selected at random for outcome assessment? In other words, were control animals and experimental animals recorded in groups?
7. Was the outcome assessor blinded? This could be either during analysis or data collection.
8. Were incomplete outcome data adequately addressed?
9. Are reports of the study free of selective outcome reporting?
10. Was the study apparently free of other problems that could result in high risk of bias?

**Table 4:**
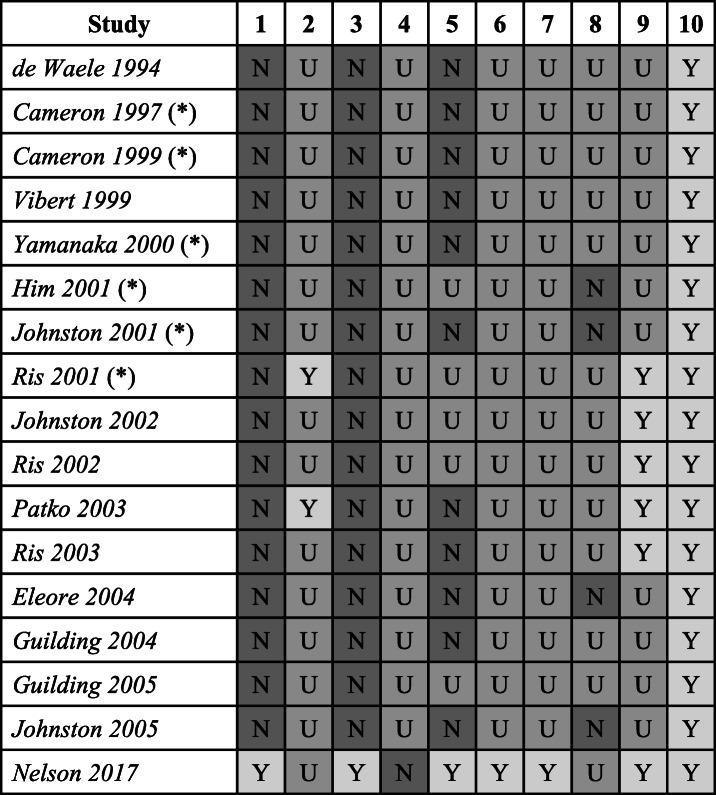
Ratings for domains assessing the risk of bias in studies included in the systematic review

Asterisked studies were included in the meta-analysis. *N* not satisfied, *U* unclear or insufficient evidence to make an assessment, *Y* criterion satisfied

### Outcome measures

The primary outcome measure for this work was the raw mean difference in spontaneous discharge rate of MVN neurons following vestibular labyrinthectomy. Secondary outcomes included changes in MVN neuronal gain, variation in ion channel expression on the MVN neuron cell membrane and modulation of sensitivity to neurotransmitters.

### Meta-analysis

The primary outcome was measured as the raw mean difference in spontaneous discharge rate between the experimental group (UVD) and the control group (either sham-operated or unoperated). This measure was used as the reported mean differences in studies were presented in a scale that was directly comparable between studies (spikes per second). Raw mean difference was calculated using the equation:

$${y}_{\mathrm{RMD}}=\overline{X_E}-\overline{X_C}$$

where y_RMD_ is the raw mean difference (referred to as the effect size), $\overline{X_E}$ is the sample mean for the experimental group and $\overline{X_C}$ is the sample mean for the control group. Outcomes were weighted using a pooled variance, which was calculated from the reported standard error of the mean (SEM) for each experiment. Pooled variance was calculated using the equation:

$${v}_{\mathrm{RMD}}={s}_{\mathrm{pooled}}^2\left(\frac{1}{n_E}+\frac{1}{n_C}\right)$$

where *v* is the approximate sampling variance, ${s}_{\mathrm{pooled}}^2$ is the pooled sampling variance across both experimental and control groups and *n*_*E*_ and *n*_*C*_ the number of recorded neurons in the experimental and control groups respectively. ${s}_{\mathrm{pooled}}^2$ is calculated from the equation:

$${s}_{\mathrm{pooled}}^2=\frac{\left({n}_E-1\right){s}_E^2+\left({n}_C-1\right){s}_C^2}{n_E+{n}_C-2}$$

The aim of the review was to determine whether UVD has an effect on the MVN neuron population as a whole. To determine an estimate of the mean effect across a population of all possible studies (*μ*), we performed a meta-analysis of the data manually in Microsoft Excel® using a random effects model. When pooling data from multiple experiments, the random effects model assumes that the observed effect in each experiment (*y*) is made up of the true effect in that study and some sampling error, which is dependent on a number of factors such as study design and execution. For example, in a pool of experiments, the model assumes that in the *i*th experiment:

$${\uptheta}_i=\upmu +{\updelta}_i$$

where θ_*i*_ is the true effect in the *i*th study, *μ* is the mean effect across a population of all possible studies and δ_*i*_ is the deviation of the *i*th study’s effect from the population mean. Here, the studies that met the inclusion criteria are considered to be a sample from the population of possible evaluations of the effect of UVD on MVN neurons. From this, the mean effect (*μ*) and population variance (Δ^2^), which is roughly equivalent to the variance of δ, can be estimated. Since this estimate is made across studies, there may be variation in the pool of experiments, termed heterogeneity. The test statistic (*Q*) can be used to assess the degree of heterogeneity between studies and incorporates the observed treatment effects and an estimate of treatment effect weighted by the observed variance. *Q* is calculated using the equation:

$$Q=\sum \limits_i^k{w}_i{\left({y}_i-\overline{y_w}\right)}^2$$

where *k* is the number of observations or studies, *y*_*i*_ is the *i*th observation of the effect of UVD, *w*_*i*_ is the inverse of the *i*th sampling variance:

$${w}_i=\frac{1}{v_{\mathrm{RMD}}}$$

and $\overline{y_w}$ is weighted estimator of treatment effect:

$$\overline{y_w}=\frac{\sum \limits_i{w}_i{y}_i}{\sum \limits_i{w}_i}$$

The test statistic *Q* approximates a χ^2^ statistic with *k* − 1 degrees of freedom [33] and can be used to test the null hypothesis *H*_0_: Δ^2^ = 0. If Δ^2^ ≠ 0, *Q* can be used as an estimate of Δ^2^, to yield a new weighted estimator *w*^∗^ that accounts for the variability in the population of studies. *w*^∗^ is calculated using the equation:

$${w}_i^{\ast }=\frac{1}{w_i^{-1}+{\Delta }_w^2}$$

where ${\Delta }_w^2$ is given by:

$${\Delta }_w^2=\frac{Q-\left(k-1\right)}{\sum \limits_iw-\sum \limits_i{w}_i^2/\sum \limits_i{w}_i}$$

This new weight is used to calculate an estimate of the average effect (μ_*w*_):

$${\upmu}_w=\frac{\sum \limits_i{w}_i^{\ast }{y}_i}{\sum \limits_i{w}_i^{\ast }}$$

and its standard error:

$$s.e.\left({\upmu}_w\right)=\sqrt{\frac{1}{\sum \limits_i{w}_i^{\ast }}}$$

Since some studies distinguished between neuron subtypes and anatomical location, the random effects model was used to account for study-specific effects (which are accounted for by the additional random effects variable) and improve the generalisability of the conclusions of the analysis. Confidence intervals (set at 95%) were calculated for each outcome measure and the estimate of the average effect μ_*w*_. Cohen’s *D* statistics were calculated as a further indication of the magnitude of the effect size [34]. In addition to *Q*, heterogeneity was estimated using the *H* statistic which describes the relative excess of *Q* over its degrees of freedom [35] and is calculated by:

$${H}^2=\frac{Q}{k-1}$$

Further, the inconsistency, *I*^2^, which describes the percentage of total variation across studies that is due to heterogeneity rather than chance [36] was calculated by:

$${I}^2=\frac{Q-\left(k-1\right)}{Q}$$

## Results

**Fig. 1:**
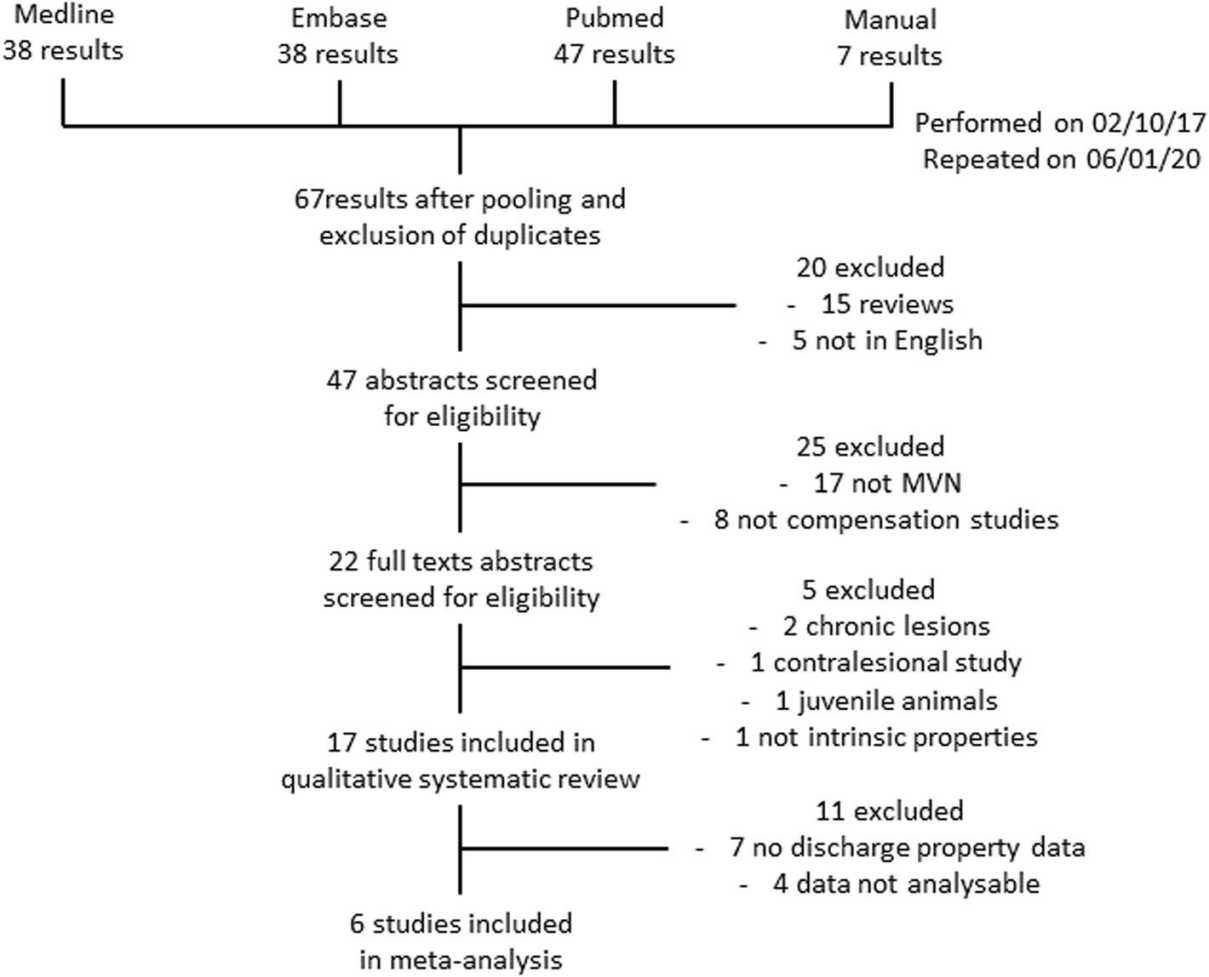
Flow chart outlining how studies were included in or excluded from the final analysis. The reasons for exclusion are detailed on the right side of the chart. This chart was generated using principles outlined in the PRISMA guideline (see main text for details)

A search of the published literature yielded 130 references. This was narrowed to 67 references after pooling and elimination of duplicates. Of these, 15 reviews and 5 references not available in English were excluded, leaving 47 relevant references. Three of the 5 references not available in English were review articles and were also excluded on this basis. English translations were found for the remaining 2 non-English articles and were excluded as they did not fulfil the study inclusion criteria. The abstracts of the remaining references were also screened and subjected to pre-specified inclusion and exclusion criteria (see the “Methods” section), leaving 22 references relevant to the posed review question. After critiquing the full text (and supplementary materials when available) of each of the remaining references, 5 more studies were excluded, leaving 17 references included in the final systematic review. Of these, 6 studies had data that was presented in sufficient detail to be comparable between studies and therefore suitable for meta-analysis (Fig. 1).

### Quality assessment

To determine the quality of the evidence, each study was assessed on three methodological domains: criterion validity of the model (Table 2), precision of experimental technique (Table 3) and risk of bias (Table 4). The details of how each assessment was made are presented in the appendix. Across all studies, there was insufficient data to make appropriate assessments for a relatively large proportion of questions. Specifically, the risk of bias was unclear in 76% of studies, the risk of imprecision was unclear in 71% of studies and the risk of model invalidity was unclear in 41%. This precluded an accurate assessment of the quality of certain studies. This suggests that there is only a small amount of evidence with relatively low risk of suffering from invalidity, bias or imprecision.

### Model validity

All studies clearly reported the animal model used and the methods employed to prepare the model for experimentation. However, a number of early experiments did not provide an explicit statement of procedures or codes used to guide safe and ethical handling of animals used in experiments. Further, 5 studies did not adequately state the routine maintenance of the model during experiments. It was unclear in 4 reports whether deafferentation was successful or confirmed. In some cases, this was implicit through references to methods sections in previous papers reporting similar experiments by the same group of experimenters.

### Precision

All studies clearly reported statistical methods used to assess significance of effects, utilised appropriate measures of variability and reported clear hypotheses to assess causality. However, there were consistent issues between studies in all domains assessed. No published study reported whether calculations of sample sizes or power required to reach significance were performed. No study clearly accounted for missing data and 5 studies had discrepancies between numbers of cells and animals published within the report raising concerns about loss of data. Only one study attempted to justify exclusion of data based on pre-specified criteria; however, the numbers of animals or cells excluded on this basis were not clearly reported. Only 2 studies reported the number of repeats of each experiment performed on a per animal and per cell basis, complicating the assessment of variability within studies.

### Risk of bias

All studies were free from obvious problems that could act as sources of bias. Only 2 studies provided enough information regarding the details of the experimental animals to ensure similar characteristics at baseline; however, no study clearly reported between-group differences. Only 1 study generated a random, concealed allocation sequence and blinded outcome assessors during data collection and analysis. Data was reported clearly for most measured outcomes studied in 6 studies, while 4 studies only reported data for certain outcomes in certain experimental groups. An assessment could not be made for many of the criteria due to the lack of relevant information presented in most reports.

Together, these assessments suggest that there is often incomplete reporting of important methodological characteristics in reports on UVD and intrinsic properties. Therefore, it is difficult to assess the quality of the evidence testing the hypothesis; however, it is generally weak based on methodological criteria alone.

### Meta-analysis

Studies presenting electrophysiological studies used either mean spontaneous discharge rates or the gradients of input-output curve functions (gains) to describe changes in intrinsic excitability following UVD. Six of the included studies [14–17, 26, 37] reported spontaneous spike discharge rates, while 2 [19, 28] reported input-output gains. Unfortunately, there was not enough numerical data within the latter 2 reports to derive spontaneous spike discharge rate measures and this data was not included in the meta-analysis.

**Fig. 2:**
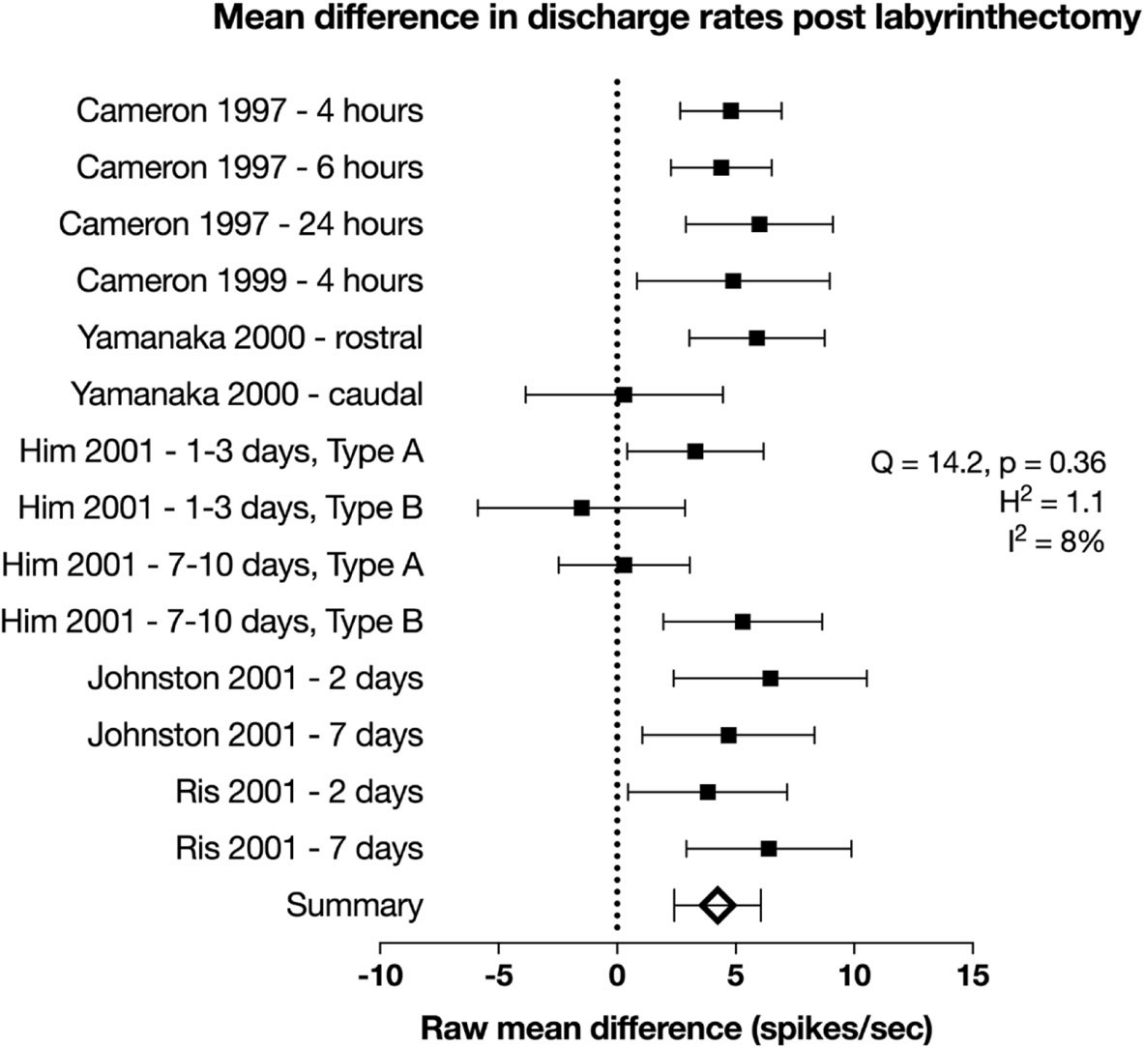
Forest plot of raw mean difference in discharge rates following labyrinthectomy observed in different studies. In the inset are calculated measures of heterogeneity. Error bars are 95% confidence intervals

Each of the 6 studies presented spike discharge rates at various time points (between 4 h and 7 days) following the lesion. One study [18] reported differences in firing between the two subtypes of MVN neuron, while another study [14] distinguished neurons based on relative anatomical location. All data sets were treated as distinct experiments and analysed separately, creating 14 individual sets of data (see the “Discussion” section for validity of this pooling). The data sets reported spike discharge rates for a total of 1216 neurons (405 control, 811 experimental) across at least 116 animals. Raw mean differences in discharge rates between experimental conditions and control were calculated and used as the effect size for the meta-analysis (Table 2). Data was plotted on a forest plot comparative purposes (Fig. 2). The majority of data sets (11 of 14, 79%) reported an increase in the mean difference between spontaneous spike discharge rates at time points up to 1 week following labyrinthectomy compared to intact or sham-operated controls (Fig. 2). The only studies reporting decreases found this was isolated to type A neurons [18] or those found in the caudal aspect of the MVN [14], a region which is thought to contain a higher proportion of type A neurons. Other studies did not explicitly distinguish between neuronal subtype or anatomical location and this difference could not be explored any further using the current data set. Using a random effects model, the mean difference in spontaneous discharge following labyrinthectomy was estimated to be 4.06 ± 1.14 (*n* = 14, 95% CI) compared to the control rate. The *Q* statistic was not significant (14.2, *p* = 0.36, χ^2^ statistic with 13 degrees of freedom) and the H statistic was 1.1, both suggesting a moderate degree of heterogeneity. However, the *I*^2^ value was 8% suggesting that a large proportion of the heterogeneity across studies is due to chance. The Cohen *D* statistic estimated the average effect size to be 0.48, consistent with a moderate size of effect. Together, this is strong evidence of a moderate increase in the intrinsic excitability of MVN neurons following UVD.

**Fig. 3:**
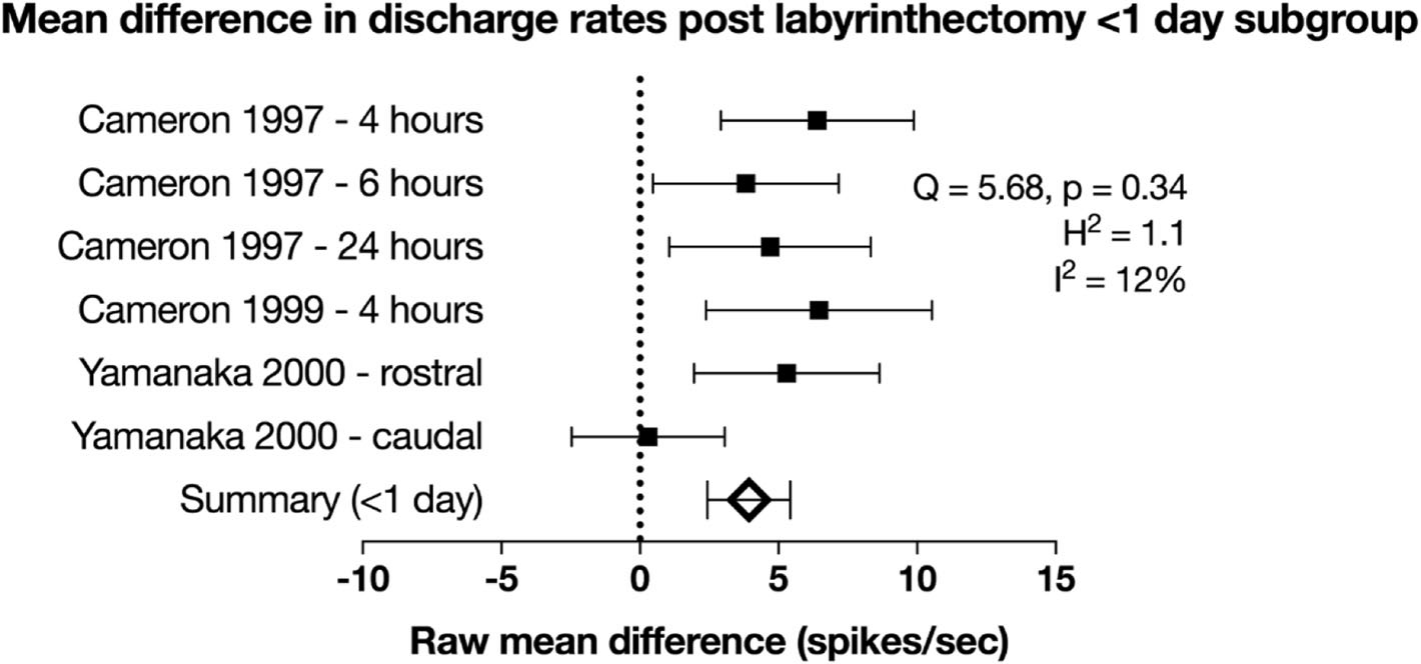
Forest plot of raw mean difference in discharge rates following labyrinthectomy performed less than 1 day prior. In the inset are calculated measures of heterogeneity. Error bars are 95% confidence intervals

Subgroup analyses were performed based on the time post UVD. Data was divided into groups of less than or equal to 1 day from lesioning or between 1 and 10 days from lesioning. For the less than or equal to 1 day subgroup, the mean difference in spontaneous discharge following labyrinthectomy was estimated to be 4.24 ± 1.81 (*n* = 6, 95% CI) compared to the control rate (Fig. 3). The *Q* statistic was not significant (5.69, *p* = 0.34, χ^2^ statistic with 5 degrees of freedom) and the *H* statistic was 1.14, suggesting a moderate degree of heterogeneity. However, the *I*^2^ value was 12% suggesting that a large proportion of the heterogeneity across studies is due to chance. The Cohen *D* statistic was 0.46, consistent with a moderate size of effect. This result is strong evidence of a moderate increase in the intrinsic excitability of MVN neurons in the acute period following UVD.

**Fig. 4:**
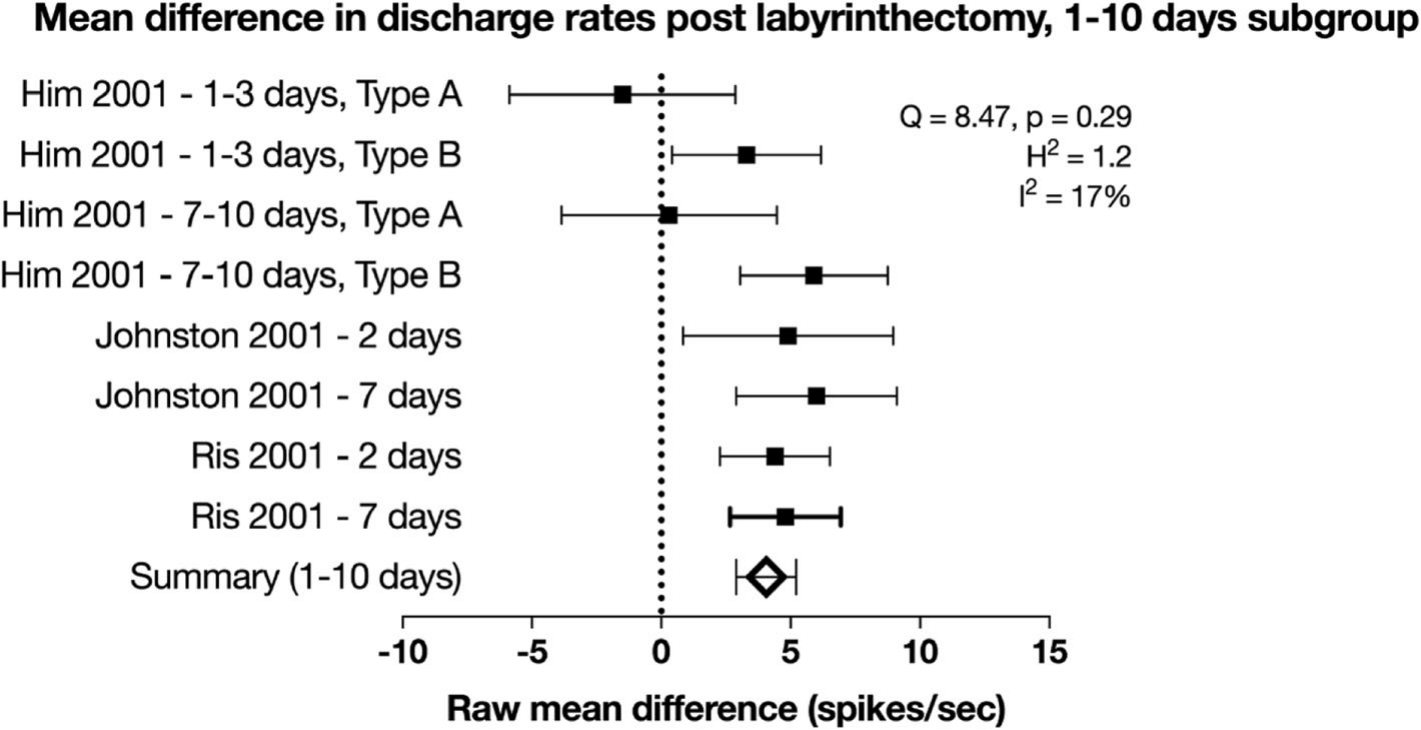
Forest plot of raw mean difference in discharge rates following labyrinthectomy performed between 1 and 10 days prior. In the inset are calculated measures of heterogeneity. Error bars are 95% confidence intervals

For the 1 to 10 days subgroup, the mean difference in spontaneous discharge following labyrinthectomy was estimated to be 3.94 ± 1.48 (*n* = 8, 95% CI) compared to the control rate (Fig. 4). The *Q* statistic was not significant (8.47, *p* = 0.29, χ^2^ statistic with 7 degrees of freedom) and the *H* statistic was 1.21, suggesting a moderate degree of heterogeneity. However, the *I*^2^ value was 17% suggesting that a large proportion of the heterogeneity across studies is due to chance. The Cohen *D* statistic was 0.49, consistent with a moderate size of effect. This is evidence that the increase in the intrinsic excitability of MVN neurons persists outside of the acute and into the subacute period following UVD.

## Discussion

We present the results of the first comprehensive, systematic review of the published literature regarding the effect of UVD on the intrinsic properties of MVN neurons. We found strong evidence that the intrinsic properties of MVN neurons change during UVD, as evidenced by an increase in their spontaneous discharge rates at rest. The magnitude of the effect is on average 4 spikes/s higher than pre-lesion rates. There is insufficient evidence to determine whether intrinsic plasticity changes differ between anatomical location or animal model.

### The role of systematic reviews of laboratory data

A systematic review integrates different sources and types of evidence to generate a summary estimate of the effect of a particular intervention or technology [38]. It derives its evidentiary power over the commonly used narrative review from its transparent and rigid methodology, designed to critically appraise included data and reduce sources of systematic bias. It is held to be one of the highest standards of evidence in medicine; however, they are rarely conducted in the analysis of primary data obtained from laboratory-based research studies [39]. In particular, meta-analysis, which allows the pooling of quantitative data to compare the effects of a particular intervention across studies [40, 41], is underutilised. The strength of the conclusions of a systematic review is ultimately dependent on the quality of the evidence base. We assessed methodological quality across three domains—precision, model validity and the risk of bias. This approach has been used in the systematic reviews of other basic experimental data [42].

### Model validity

The models chosen for the assessment of the study question were very similar across studies. All studies used appropriate animals (mouse, rat, guinea pig) that are known to experience a resolution in static symptoms following vestibular compensation within a week. Most experiments performed unilateral labyrinthectomies, while one study utilised a more rigorous sham-operated control. Technically, a sham operation is the most appropriate control and should be considered the gold-standard control for future studies; however, the ethics of this need to be considered [43, 44]. Three studies [28, 30, 31] were assessed to have a high risk of model invalidity due to a lack of reporting of routine model maintenance. In particular, there were no details of housing or ages of animals (or a surrogate such as weight) that may influence experience-dependent changes. Reporting of whether the success of the procedure to achieve UVD was confirmed was not consistent across these three studies; however, references to previous work were made suggesting that the chosen methods were likely appropriate.

### Precision

One major issue in this assessment of precision was distinguishing between technical and observer variability. The former is a function of the number of repeats performed within each animal, attesting to potentially modifiable issues with recording techniques. Meanwhile, the latter is dependent on the number of animals used for all experiments and reveals more random uncontrollable differences between animals. In all included studies, recordings were made from multiple individual neurons in multiple animals. However, there was no reporting of comparisons between animals, nor was there consistent reporting of the number of slices created from each animal. All reports presented results as a single pool of recorded neurons, such that measures of variability were probably a combination of technical and observer variability, precluding an assessment of these domains independently. This raises the concern of pseudoreplication [45]. The question posed in most studies was whether UVD changed the intrinsic excitability of MVN neurons. This question can be answered by using the neuron as the experimental unit, as was done in each of the included studies. However, this does not address whether there are between-animal differences in the effect of UVD that have not been explicitly addressed. This domain can be accounted for using multi-level statistical models that take into account this animal factor during analysis. Alternatively, an experiment can be performed whereby the animal is the experimental unit and neurons from each animal are pooled into an average [46]. This would have to be appropriately powered with a prior calculation of sample sizes to ensure sufficient animals to achieve a meaningful result.

### Meta-analysis

Meta-analysis is a powerful tool for averaging effects across multiple studies. The main benefit of this procedure over arithmetic averaging is the systematic evaluation of heterogeneity between studies and the weighting of studies based on the degree of observed variation. In this pool of data, there was very little calculated heterogeneity between studies as calculated by the H statistic, which is particularly effective for analyses of more than 8 studies [35]. A question remains as to whether it is appropriate to consider MVN neurons as a uniform neuronal subtype. There is evidence that, at least chronically, MVN neuronal subtypes ipsilaterally appear to become homogenised, approaching a more type A-like profile with linear characteristics [47]. However, this work has not been conducted in the acute stage. Studies did not consistently differentiate between neuronal subtype and therefore it is not possible to make a conclusive determination of the validity of this pooling.

### Limitations

There are a number of limitations in this approach. The search strategy used to find evidence revealed a very large number of studies. Using more restrictive search terms, searches revealed less reports that may have potentially excluded some relevant studies. For example, phrases ‘damage’ and ‘lesion’ revealed many more irrelevant studies than using the more specific terms ‘deafferentation’ and ‘labyrinthectomy’. However, some reports used the word ‘damage’ in their title and abstract to generalise their findings and were only found by a manual search of references from other papers. Further, certain phrases are not used consistently between studies to describe the changes they observed. A number of reports describe either increase in spontaneous discharge or firing rate and do not describe this consistently as a change in ‘intrinsic excitability’. These inconsistencies in vocabulary within the field could skew the range of evidence found by the search strategy; however, all efforts were made to be as inclusive as possible.

Some studies presented all data graphically while reporting only numerical values for positive results. This precluded some of the results that demonstrated no change following UVD from being included in the meta-analysis. This potentially skews the results towards a false-positive assessment. Further, this prevented the analysis of data from 2 studies which were of high methodological quality, reducing the power of the meta-analysis. Incomplete reporting should therefore be avoided in the future to permit such analyses that integrate findings across studies and may potentially help reduce publication bias.

## Conclusions

There is a corpus of evidence demonstrating that UVD increases neuronal excitability. Using the systematic review and meta-analysis technique, we conclude that this evidence is robust and concordant, lending strong support to the hypothesis that intrinsic mechanisms play a role in vestibular compensation. Our analysis also highlights the inherent utility of collating and pooling data from disparate sources to enhance the strength of assertions made through laboratory-based experiments.

## Data Availability

All data used in the preparation of this manuscript were sourced from the original research publications. The availability of the original datasets is subject to release by the original authors.

## Abbreviations

MVN: Medial vestibular nucleus
PRISMA: Preferred Reporting Items for Systematic Reviews and Meta-Analyses
SYRCLE: Systematic Review Centre for Laboratory Animal Experimentation
UVD: Unilateral vestibular deafferentation
VOR: Vestibuloocular reflex
